# Involvement of ethylene biosynthesis and signalling in fruit set and early fruit development in zucchini squash (*Cucurbita pepo* L.)

**DOI:** 10.1186/1471-2229-13-139

**Published:** 2013-09-22

**Authors:** Cecilia Martínez, Susana Manzano, Zoraida Megías, Dolores Garrido, Belén Picó, Manuel Jamilena

**Affiliations:** 1 Departamento de Biología y Geología, Agrifood Campus of International Excellence (ceiA3), Universidad de Almería, La Cañada de San Urbano s/n, 04120 Almería, Spain; 2 Departamento de Fisiología Vegetal, Universidad de Granada, Fuentenueva s/n, 18071 Granada, Spain; 3 Departamento de Biotecnología, Universidad Politécnica de Valencia, Valencia, Spain

**Keywords:** *Cucurbita pepo*, Fruit set, Parthenocarpy, Ethylene, Gene expression, Auxin

## Abstract

**Background:**

We have identified a kind of parthenocarpy in zucchini squash which is associated with an incomplete andromonoecy, i.e. a partial conversion of female into bisexual flowers. Given that andromonoecy in this and other cucurbit species is caused by a reduction of ethylene production in the female flower, the associated parthenocarpic development of the fruit suggested the involvement of ethylene in fruit set and early fruit development.

**Results:**

We have compared the production of ethylene as well as the expression of 13 ethylene biosynthesis and signalling genes in pollinated and unpollinated ovaries/fruits of two cultivars, one of which is parthenocarpic (Cavili), while the other is non-parthenocarpic (Tosca). In the latter, unpollinated ovaries show an induction of ethylene biosynthesis and ethylene signal transduction pathway genes three days after anthesis, which is concomitant with the initiation of fruit abortion and senescence. Fruit set and early fruit development in pollinated flowers of both cultivars and unpollinated flowers of Cavili is coupled with low ethylene biosynthesis and signalling, which would also explain the partial andromonoecy in the parthenocarpic genotype. The reduction of ethylene production in the ovary cosegregates with parthenocarpy and partial andromonoecy in the selfing progeny of Cavili. Moreover, the induction of ethylene in anthesis (by ethephon treatments) reduced the percentage of bisexual parthenocarpic flowers in Cavili, while the inhibition of ethylene biosynthesis or response (by AVG and STS treatments) induces not only andromonoecy but also the parthenocarpic development of the fruit in both cultivars.

**Conclusions:**

Results demonstrate that a reduction of ethylene production or signalling in the zucchini flower is able to induce fruit set and early fruit development, and therefore that ethylene is actively involved in fruit set and early fruit development. Auxin and TIBA treatments, inducing fruit set and early fruit development in this species, also inhibit ethylene production and the expression of ethylene biosynthesis and response genes. A model is presented that discusses the crosstalk between ethylene and auxin in the control of fruit set and early fruit development in zucchini squash.

## Background

Despite its molecular simplicity, ethylene regulates a number of developmental and physiological processes [1,2], including leaf and flower abscission, ripening of climacteric fruit and biotic and abiotic stresses. In the species of the *Cucurbitaceae* family ethylene controls sexual expression and is the main determinant of sexual phenotypes [3-5]. Thus, the ethylene biosynthesis genes *CmACS7* and *CsACS2* of melon and cucumber, respectively, regulate the arrest of stamen development in female flowers of monoecious cultivars, and their loss of function mutations lead to the conversion of female into bisexual flowers, and therefore the transformation of monoecious into andromonoecious cultivars [5-7]. In *Cucurbita pepo*, ethylene also regulates the sexual expression of monoecious cultivars, controlling both the precocity and the number of female flowers [4,5,8,9]. In fact, the application of blocking agents of ethylene production (AVG) or perception (STS) is able to delay female flowering and reduce the number of female flowers per plant, but also to induce a conversion of female into bisexual flowers [4]. Likewise, female flowers of zucchini produce much more ethylene than male flowers throughout their development and maturation up to anthesis [4].

After pollination and fertilization, fruit set and fruit development is dependent on cell division and expansion promoted by hormones such as gibberellin (GAs), auxin and cytokinin [10-12]. Auxin is the determinant of fruit set, as has been demonstrated by the study of mutants or transgenic lines for *ARF* or *IAA/Aux* multigene families in tomato and *Arabidopsis*[10-12]. However, they seem to be closely related to gibberellin which is able to trigger fruit initiation without changes in auxin signalling genes [13,14]. In the *Cucurbitaceae* family, fruit set and development depend mainly on auxin [15]. The application of auxins induces parthenocarpic fruit set and development in cucumber [16], although the application of cytokinin also activates cell divisions in fruits [17], whereas brassinosteroid increase fruit set [10,18]. In zucchini auxin is also shown to be the most effective hormone to induce parthenocarpic fruit development [19], and this growth regulator is commonly applied to promote fruit set and growth in greenhouse production of this vegetable crop.

Ethylene has been related with floral organ senescence and abscission after pollination. Pollination induces ethylene production in the ovaries and petals, and this ethylene appears to be responsible for coordinating ovary growth and petal senescence [20-23]. The implication of ethylene in fruit set and development was not studied in depth until a few years ago. Recent studies have shown an interconnection between early ovule abortion and the size of the silique in *Arabidopsis* ethylene mutants [24]. Meanwhile, pollination and gibberellin treatments are responsible for downregulating ethylene biosynthesis and signalling genes in tomato immediately after fruit set [12,22].

Since *C. pepo* morphotype zucchini has a large inferior ovary (about 6–8 cm long at anthesis), it is an ideal species suitable to study fruit set and early fruit development. We have recently observed that a reduction of ethylene in female flowers of zucchini can not only promote the development of stamens in the flower, converting female into bisexual flowers, but also induce the parthenocarpic fruit development in absence of pollination and fertilization [25]. This is also true for some cultivars of zucchini squash grown under high temperature conditions [25]. To study the role of ethylene in fruit set and early fruit development in zucchini squash, the present paper compares the production of ethylene and the expression of ethylene biosynthesis and response genes between pollinated and unpollinated ovaries of a non-parthenocarpic cultivar, as well as between a parthenocarpic and a non-parthenocarpic cultivar of zucchini squash. Moreover, we analysed fruit growth rates as well as ethylene production and the expression of ethylene genes in response to auxin and TIBA treatments. Results indicate that ethylene is directly involved in fruit set and early fruit development in this species. These two developmental processes require a low level of ethylene production and signalling within the few days after pollination, and the loss of pollination and fertilization is accompanied by an induction of ethylene biosynthesis and signalling 3 days after anthesis, concomitantly with fruit abortion.

## Results

Different approaches have been used to determine the implication of ethylene in zucchini fruit set and early fruit development. Firstly we have determined ethylene production and the expression of 13 ethylene biosynthesis and signalling genes in pollinated and unpollinated ovaries and fruits of the non-parthenocarpic cultivar Tosca. Secondly, we have compared ethylene production and the expression of ethylene genes in the fruits of two contrasting cultivars for parthenocarpy: Tosca and Cavili. Finally, the same two cultivars were used to study the crosstalk between ethylene and auxins in the control of these developmental processes.

### Ethylene production and ethylene genes expression in pollinated and unpollinated ovaries/fruits of zucchini

**Figure 1:**
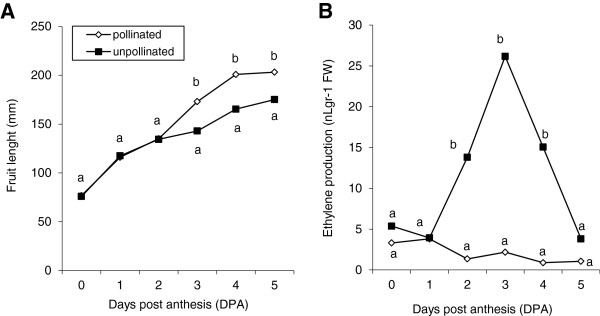
**Evolution of the size and ethylene production in pollinated and unpollinated ovaries/fruits of zucchini cv. Tosca. (A)** Fruit size. **(B)** Ethylene production. Different letters indicate significant differences between the means at each data point between pollinated and unpollinated ovaries/fruits (p ≤ 0.05; n = 12 for fruit length, and n = 4 for ethylene production).

It has been shown that the fruits of many zucchini cultivars can initiate their growth in the absence of pollination and hormone application. In fact, in the non-parthenocarpic cv. Tosca the growth rate of pollinated and unpollinated fruits were very similar for the first three days (Figure 1A), which highlights the natural parthenocarpy of this species. After the third day, however, most unpollinated fruits aborted, and some of them grew at a significantly slower rate than pollinated fruits (Figure 1A). In the first 5 DPA, the profiles of ethylene production in pollinated and unpollinated fruits were very dissimilar (Figure 1B). While ethylene production decreased slightly in pollinated fruits, in unpollinated fruits it increased sharply 3 DPA (Figure 1B). These results indicate that the decrease in the growth rate of unpollinated fruits that occurs at 3 DPA is correlated with a burst of ethylene in the fruit, and that the maintenance of fruit growth likely requires a low level of ethylene.

**Figure 2:**
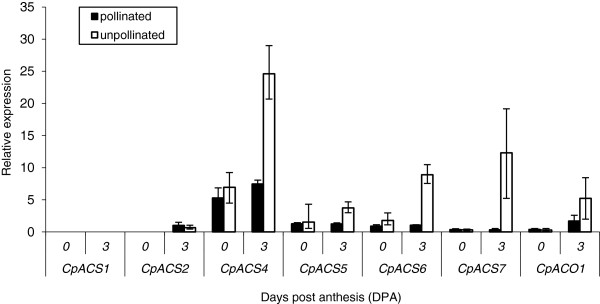
**Relative expression of 7 ethylene biosynthesis genes in pollinated and unpollinated ovaries/fruits of zucchini cv. Tosca at anthesis (0 DPA) and 3 DPA.** In pollinated flowers, expression at the day of anthesis was determined 8 h after hand pollination. Each data point represents the mean of 3 replicates with 3 fruits each. Error bars indicate SE.

To detect putative genes regulating ethylene biosynthesis during fruit set and early fruit development, we have analysed the expression of six *ACS* genes and one *ACO* gene from *C. pepo* (Additional file 1: Table S1, Figure 2). *CpACS1* was one of the first *ACS* genes isolated from plants [3]; *CpACS2*, and *CpACS4* to *CpACS6* are four unigenes identified by new generation sequencing (NGS) of cDNA from different squash tissues [26]; available at Cucurbigene.net]; and *CpACS7* and *CpACO1* have been isolated by PCR strategy with degenerated primers (unpublished). Expression was studied through qPCR at anthesis (0 DPA) and 3 DPA (Figure 2). *CpACS1* was not expressed during this period (Figure 2). At anthesis, the expression of the other 6 genes was very low, except for *CpACS4*, which could explain the basal production of ethylene at this point. This basal expression was maintained in pollinated fruits at 3 DPA (Figure 2). Nevertheless, in unpollinated fruits the expression of the five *ACS* genes and *CpACO1* was upregulated at 3 DPA. The highest induction was observed in *CpACS4*, *CpACS6* and *CpACS7* (Figure 2), which appear to be the main gene responsible for the ethylene produced at this point.

**Figure 3:**
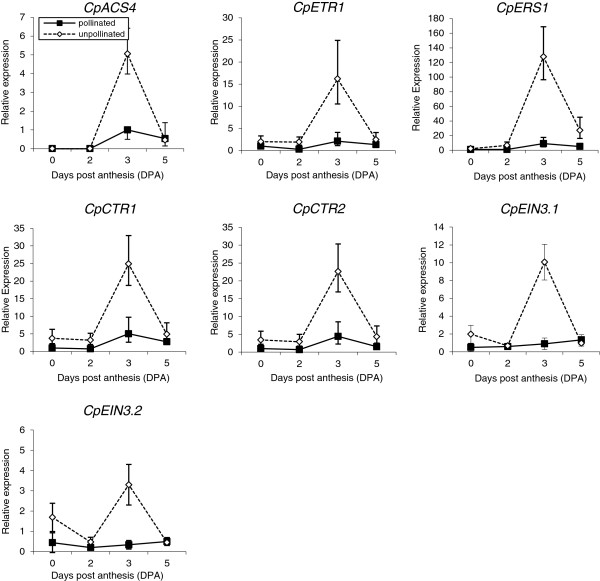
**Relative expression of ethylene biosynthesis (*CpACS4*), perception (*CpETR1 *and *CpERS1*) and signalling (*CpCTR1*, *CpCTR2*, *CpEIN3.1 *and *CpEIN3.2*) genes over 5 DPA in pollinated and unpollinated ovaries/fruits of cv. Tosca.** Each data point represents the mean of 3 replicates with 3 ovaries/fruits each. Error bars indicate SE.

To determine the molecular mechanisms behind the action of ethylene in fruit set and early fruit development, the relative expressions of six additional ethylene perception and signalling genes were also studied in pollinated and unpollinated fruits over 5 DPA. As occurred for *CpACSs* and *CpACO1*, other genes involved in ethylene perception such as *CpETR1* and *CpERS1*, as well as in ethylene signalling (*CpCTR1*, *CpCTR2*, *CpEIN3.1* and *CpEIN3.2*), also showed an expression profile similar to that of ethylene production (Figure 3). In pollinated fruits, expression levels remained low during early development, while in non-pollinated fruits, all perception and response genes were significantly upregulated at 3 DPA (Figure 3), coinciding with the peak of ethylene and the abortion of fruit growth (Figure 1).

### Ethylene is actively involved in fruit set and early fruit development in zucchini

In order to ascertain whether the differences in ethylene production between pollinated and unpollinated fruits at 3 DPA are the consequence of fruit set or fruit abortion, or rather a signal that is actively involved in fruit set and development, we have compared ethylene production and signalling in one parthenocarpic and one non-parthenocarpic variety, as well as in a segreganting population derived from the parthenocarpic one, and determined the effects of ethylene releasing and blocking agents on fruit set and early fruit development.

**Figure 4:**
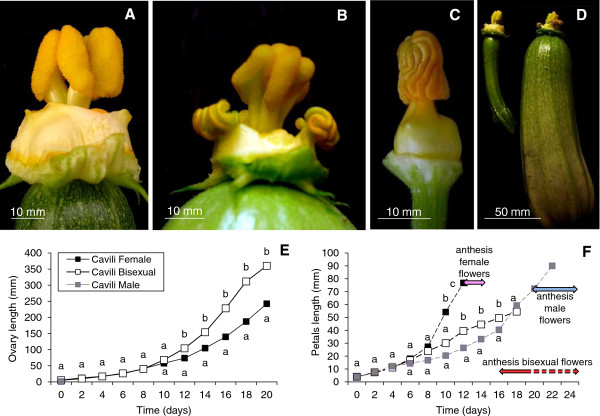
**Growth rates of ovaries/fruits and petals in female, bisexual and male flowers of the parthenocarpic cultivar Cavili. (A)** Female flower. **(B)** Bisexual flower with a partial development of stamen. **(C)** Male flower. **(D)** Ovary size of female and bisexual flowers with the same time of development. Flowers were tagged when they were 4 mm in length (time 0), and allowed to grow for 20 days in the case of female and bisexual flowers, and 24 days in the case of male flowers. **(E)** Comparison of ovary/fruit longitudinal growth rates between female and bisexual flowers. **(F)** Comparison of petal longitudinal growth rates of female, bisexual and male flowers. Note that petal growth is delayed in bisexual flowers, but not as much as in male flowers. Anthesis in the bisexual flowers is delayed even more than in male flowers. Statistical analysis was performed using the LSD method (p ≤ 0.05; n = 15).

The hybrid cultivar Cavili is able to develop parthenocarpic fruits of commercial size in absence of pollination or hormonal treatments [25]. The parthenocarpy of this cultivar is associated with an incomplete andromonoecy, i.e. a partial conversion of female into bisexual flowers, and a delay in floral organ maturation (Figure 4). Ovary-bearing flowers of this cultivar can be classified into female flowers with no stamen development (Figure 4A), or bisexual flowers showing a certain degree of stamen development (Figure 4B), but never reaching the size of stamen in male flowers (Figure 4C). At the same stage of development, bisexual flowers always showed a higher ovary and fruit size (Figure 4D). We compared the longitudinal growth rate of ovaries/fruits between female and bisexual flowers for a total of 22 days, starting with floral buds of about 4 mm in length (Figure 4E and F). The growth rate of ovary length in bisexual flowers deviated from that of female flowers at twelve days, immediately after anthesis of female flowers (Figure 4F). By this time petals of bisexual flowers were still immature and closed but their ovaries kept growing at a much faster rate than those of female flowers. Many of the ovaries in bisexual flowers reached a commercial size before anthesis. In fact, many of the bisexual flowers did not reach anthesis in the 24 days of study, and in others anthesis was delayed with respect to female flowers because of a lower growth rate of petals (Figure 4F). These results indicate that the parthenocarpy of this cultivar is not only correlated with stamen development, but also with a lower growth rate of petals, which delays maturation of petals and anthesis. Given that male flowers require twice as long as female ones to mature and reach anthesis (Figure 4F), it is likely that the delay in the maturation of bisexual flowers is associated with their masculinisation, i.e. the presence of stamens.

To assess whether parthenocarpy and partial andromonoecy had the same genetic regulation, we phenotyped the F2 population derived by self-pollination of the F1 hybrid Cavili, which segregates for the two traits. A complete cosegregation between the two traits has been found in the F2 generation. Of a total of 95 plants, 23 were completely monoecious, and produced only female flowers, while 72 were partially andromonoecious and produced both female and bisexual flowers (Table 1). Although the number of bisexual flowers in the latter varied from 20% to 100%, all of them developed into parthenocarpic fruits, which reached commercial size even before anthesis (Table 1). The 3:1 segregation ratio (χ^2^ = 0,014, p value = 0,91) indicated that the partial andromonoecy and parthenocarpy of Cavili appears to be controlled by at least one dominant gene.

**Table 1:** Fruit size and ethylene production of female and bisexual flowers among monoecious and partially andromonoecious plants in the selfing progeny of Cavili (F2 population)

**F2 segregation (No. plants)**	**Flower phenotype**	**Ovary length at anthesis (mm)**	**Ethylene production at 3 DPA (nL/gr FW)**
Monoecious (23 plants)	Female	78.10±4.23 a	10.90±2.94 a
Partially andromonoecious (72 plants)	Female	77.43±1.00 a	10.90±2.94 a
	Bisexual	139.07±4.75 b	2.51±1.23 b

Ovary size and ethylene production data were obtained from 25 replicates for each flower phenotype. Different letters within the same column indicate significant differences between female and bisexual flowers (t-analysis, p<0,01).

**Figure 5:**
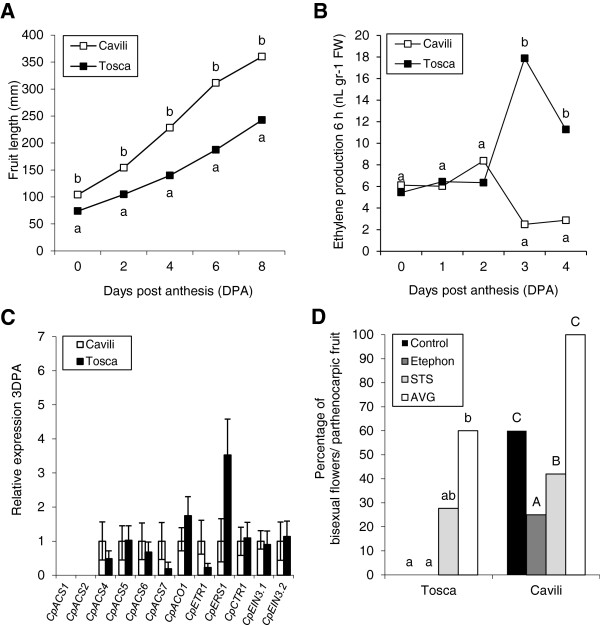
**Involvement of ethylene in the parthenocarpic development of Cavili fruit. (A)** Comparison of ovary/fruit growth between the parthenocarpic cv. Cavili (bisexual and unpollinated flowers) and the non-parthenocarpic cv. Tosca (female and unpollinated flowers). **(B)** Comparison of ethylene production in unpollinated ovaries/fruits of Cavili and Tosca **(C)** Expression of ethylene biosynthesis, perception and response genes in unpollinated ovaries of Cavili and Tosca at 3 DPA. **(D)** Effects of ethylene releasing (ethephon) and blocking reagents (AVG and STS) on the percentage of bisexual parthenocarpic flowers in Cavili and Tosca. Different letters indicate statistical differences between treatments within each cultivar (p ≤ 0.05; n = 15; lowercase Tosca; uppercase, Cavili).

Sex determination and female flower maturation in zucchini is known to be regulated by ethylene in the earliest stages of flower development [4,8,9]. Therefore, the parthenocarpy of Cavili could be the result of a reduction of ethylene in female flowers. We have found that the unpollinated Cavili ovaries/fruits produce significantly less ethylene than those of the non-parthenocarpic Tosca during the days immediately after anthesis (Figure 5B). The production of ethylene was also measured in the ovaries of 25 female and 25 bisexual sample flowers derived from the F2 population of Cavili. Results indicated that the higher growth rate of fruits in bisexual flowers cosegregated with a significant reduction of ethylene production in the ovary at 3 DPA (Table 1).

All the analyzed *ACS* genes, including *CpACS4*, *CpACS6* and *CpACS7*, which were those that mainly regulate ethylene production in unpollinated ovaries in the days immediately after anthesis, showed no significant differential expression between Cavili and Tosca unfertilized ovaries at 3DPA or showed higher expression in Cavili (Figure 5C). Only the expression of *CpACO1* was lower in Cavili (Figure 5C). It appears therefore that the reduction in ethylene production observed in the unpollinated ovary of the parthenocarpic cultivar Cavili is not regulated at the level of transcription. For ethylene perception and response genes, only the transcripts of *CpERS1* showed a lower accumulation of transcripts in Cavili (Figure 5C), suggesting that perception of ethylene could also be altered in this parthenocarpic cultivar.

To confirm whether a reduction in ethylene production during the development of female flowers was enough to induce the parthenocarpic development of the zucchini ovary, we determined the effects of ethylene releasing and blocking agents on early fruit development. Control plants of Tosca produced no bisexual parthenocarpic flowers, while those of Cavili produced 60% (Figure 5D and Additional file 2: Figure S1). The application of ethephon significantly reduced the production of bisexual parthenocarpic flowers in Cavili, while the application of the ethylene blocking agents STS and AVG increased the production of bisexual parthenocarpic flowers not only in the parthenocarpic Cavili, but also in Tosca (Figure 5D and Additional file 2: Figure S1). These results demonstrate that ethylene is actively involved in fruit set and early fruit development in zucchini squash, and indeed a reduction of either ethylene biosynthesis or signalling in the developing female flower of zucchini can not only inhibit the arrest of stamens, promoting the conversion of female into bisexual flower, but also to induce the parthenocarpic development of the fruit.

### Effects of auxins on fruit growth rates and ethylene biosynthesis and signalling

**Figure 6:**
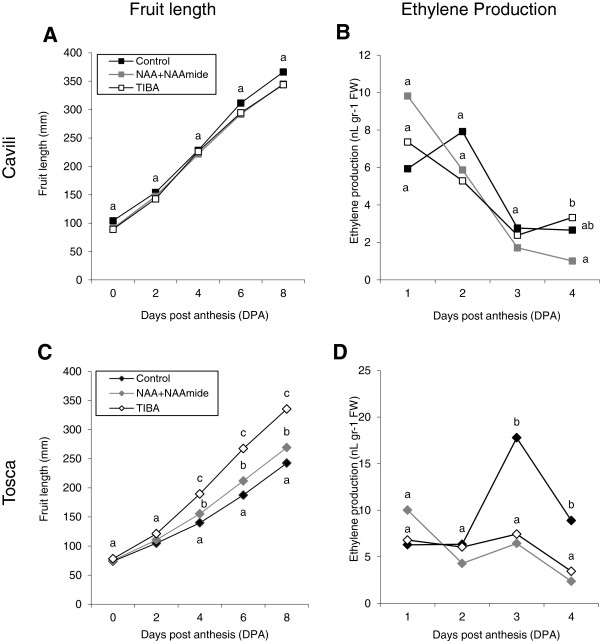
**Effects of NAA + NAAmide and TIBA on fruit growth rates and the evolution of ethylene production in Cavili and Tosca. (A)** Fruit growth rates in Cavili. **(B)** Ethylene production in Cavili. **(C)** Fruith growth rates in Tosca. **(D)** Ethylene production in Tosca. Ovaries were treated at anthesis. Different letters at the same time point indicate significant differences between treatments (p ≤ 0.05; n = 15 for ovary growth and n = 4 for ethylene production).

It is known that external application of auxins and TIBA, the latter an inhibitor of auxin polar transport, can induce fruit set and early fruit growth in different species. In zucchini, synthetic auxins are commonly used to stimulate the parthenocarpic development of fruit in off-season greenhouse production. We have studied the effects of NAA + NAAmide and TIBA (applied at anthesis in the ovary) on fruit development and ethylene production in the cultivars Cavili and Tosca. In the parthenocarpic cv. Cavili neither auxins nor TIBA were able to alter the longitudinal growth rate of the fruit (Figure 6A), suggesting that the ovaries of this cultivar could have a high concentration of auxins. However, in the fruits of the non-parthenocarpic cv. Tosca both treatments promoted longitudinal growth of the fruit, although TIBA was more effective than NAA + NAAmide (Figure 6C). Ethylene production in control and treated fruits was negatively correlated with early fruit growth rate. In Cavili, ethylene decreased progressively throughout the first 4 DPA in both control and treated fruits (Figure 4B), while in Tosca the only fruits where ethylene was induced at 3 DPA were the unpollinated control fruits, which were those which showed the lowest growth rate and finally aborted (Figure 6D).

**Figure 7:**
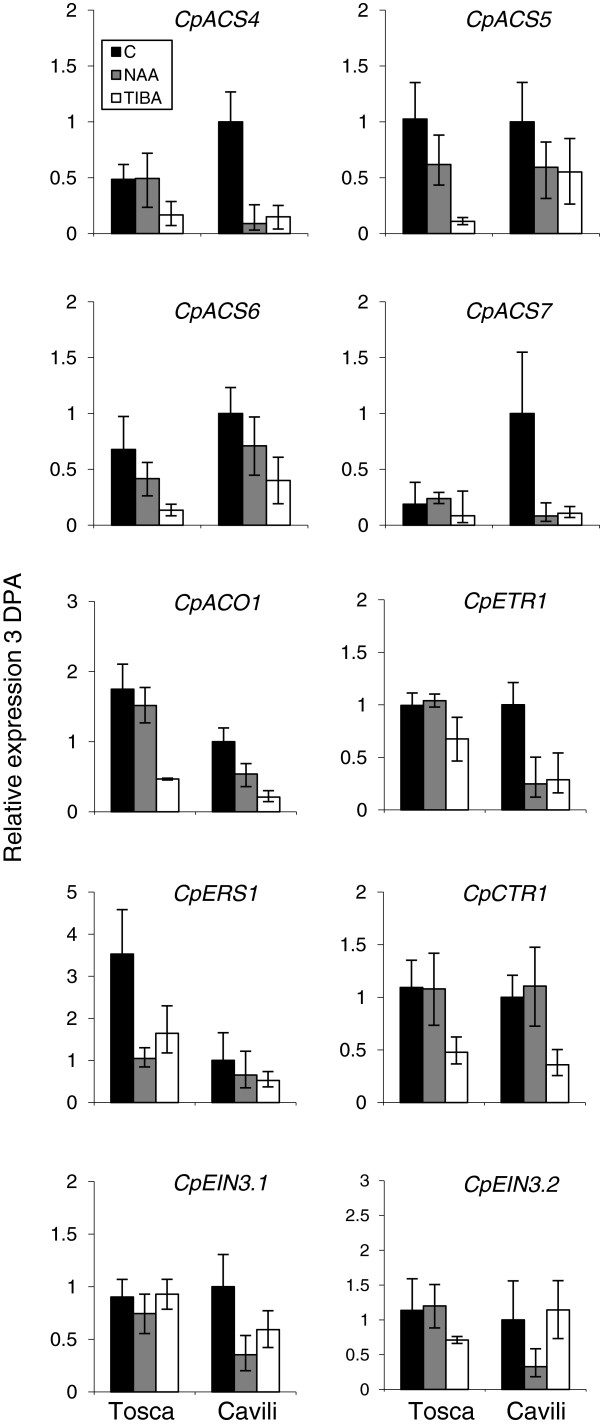
**Effect of NAA and TIBA on the expression of ethylene genes.** Flowers were treated at anthesis, and the expression of ethylene biosynthesis, perception and signalling genes was determined in the ovaries/fruit at 3 DPA, when ethylene production is induced in unpollinated ovaries. Each data point represents the mean of 3 replicates with 3 ovaries/fruits each. Error bars indicate SE.

The effect of NAA and TIBA on the expression of ethylene genes was studied in fruits at 3 DPA, when differences in ethylene production were evident between pollinated and unpollinated fruits, and between parthenocarpic and non-parthenocarpic cultivars. In concordance with ethylene production data, *ACS* and *ACO* genes were downregulated by NAA and TIBA in the fruits of both Tosca and Cavili at 3 DPA (Figure 7), indicating that auxins regulate negatively the production of ethylene in the fruit during the days immediately after anthesis. TIBA treatment was more effective than treatment with NAA + NAAmide in reducing the expression of ethylene biosynthesis genes (Figure 7). Moreover, the downregulation of *CpACS4* and *CpACS7*, the genes which contribute most to the production of ethylene in unpollinated fruits during the days immediately after anthesis, was higher than that observed for *CpACS5*, *CpACS6* and *CpACO1* (Figure 7). The expression of the ethylene receptor *CpETR1* was similar in control ovaries of Tosca and Cavili, and was significantly reduced in response to both treatments in Cavili, and in response to TIBA in Tosca. The expression of the other receptor gene *CpERS1* was higher in the non-parthenocarpic cultivar Tosca, and it was downregulated by NAA and TIBA in this cultivar (Figure 7). Regarding ethylene response genes, the expression of *CpCTR1* was also downregulated by TIBA in the two cultivars (Figure 7), but not by NAA; and although the hormonal treatments did not significantly change the expression of *CpEIN3.1* and *CpEIN3.2* in Tosca (Figure 7), in Cavili NAA downregulated the expression of both, whereas TIBA only reduced the expression of the former. In conclusion, many of the analysed ethylene biosynthesis, perception and signalling genes were downregulated by NAA and TIBA, two treatments that induce the growth rate of the zucchini fruit in the days immediately after anthesis.

## Discussion

### Fruit set and early fruit development is correlated with a downregulation of ethylene biosynthesis and signalling genes

Ethylene production and expression data from 13 genes covering ethylene biosynthesis and signal transduction pathway in *C. pepo* have demonstrated that the loss of pollination in a non-parthenocarpic genotype of this species is accompanied by a sharp increase in ethylene biosynthesis and signalling in the ovary at 3 DPA. This is true for the 5 *ACS*-like genes showing expression in the ovary (one of the analysed genes, *CpACS1*, showed no expression in the ovary during this time of development) and *CpACO1*, as well as for two ethylene receptors (*CpETR1* and *CpERS1*), two *CTR1*-like genes (*CpCTR1* and *CpCTR2*) and two *EIN3*-like genes (*CpEIN3.1* and *CpEIN3.2*). This ethylene signalling in the ovary few days after anthesis is associated with a decline in growth and finally with the abortion of fruit. Pollinated flowers, on the other hand, maintain a very low level of ethylene biosynthesis and signalling in the fruit during the days immediately after anthesis, concomitantly with fruit set and development. This lower ethylene signalling is also observed in parthenocarpic fruits of the zucchini cultivar Cavili, as well as in the parthenocarpic plants of the segregant selfing progeny of Cavili. Taken as a whole, these data indicate that the production of ethylene in the ovary few days after anthesis is negative correlated with fruit set and growth.

Although the function of ethylene in fruit set and early fruit development has not been studied in depth, it has been reported that the inhibition of ethylene production or response by external treatments with AVG, STS or 1-methylcyclopropene (1-MCP), induces fruit set in pear [27], mango [28], mandarin [29], and *Arabidopsis*[24], among others. Moreover, transcriptome analysis of tomato has recently revealed that many ethylene genes are downregulated at 3DPA in parthenocarpic, pollinated and GA_3_ treated carpels, suggesting a role of ethylene in tomato fruit set and development [12,22,30]. In this paper we have demonstrated that the inhibition of ethylene biosynthesis (AVG treatment) or response (STS treatment) is sufficient to induce the set and early development of the fruit in absence of pollination in both the parthenocarpic and the non-parthenocarpic cultivar. Our data therefore not only indicate a clear correlation between low production/signalling of ethylene and fruit set, but also demonstrate that ethylene is directly involved in fruit set and early fruit development.

Ethylene genes are highly expressed in the ovary of tomato flowers at anthesis, especially in parthenocarpic genotypes [12], but they are downregulated after fruit set, which suggests that ethylene acts as an antagonist to auxins in fruit set, preventing carpels developing into fruit before pollination and fertilization occur [22]. In zucchini, ethylene biosynthesis and signalling genes are induced in developing carpels throughout flower development and maturation up to anthesis but the level of ethylene production in anthesis is lower than that of tomato [unpublished results, [9]. Therefore, after anthesis the zucchini pollinated ovary maintains its production of ethylene (or it may fall slightly) as well as the expression of ethylene biosynthesis and signal transduction pathway genes. These differences in ethylene gene expression between tomato and zucchini carpel after anthesis could be associated with dissimilar early fruit development in tomato and zucchini. In fact, the tomato fruit does not initiate its development until pollination and fertilization occurs, while in zucchini we have observed that ovaries of pollinated and unpollinated flowers grow at the same rate during the first 3 DPA, and it is only after this point when unpollinated fruits reduced their growth and finally abort. It appears therefore that early fruit development requires a low level of ethylene biosynthesis and signalling, and that this level is already low enough in the zucchini ovary at anthesis, but it has to decrease in tomato during the first days after anthesis. The lower production of ethylene in the parthenocarpic fruits of Cavili, compared to those of the non-parthenocarpic cv. Tosca, and the cosegregation of andromonoecy, parthenocarpy and lower ovary ethylene production in the F2 generation of Cavili in the days immediately after anthesis, also support this conclusion.

The induction of ethylene biosynthesis and signalling genes in the zucchini ovaries at 3 DPA constitutes a positive signal that could be involved in the abortion and senescence of unfertilized ovaries. In zucchini, we have observed that the development of the ovary and the fruit is initiated before the occurrence of pollination and fertilization, and that the fruit continues its development if ethylene is not induced. Both pollination/fertilization and parthenocarpy may prevent the induction of ethylene biosynthesis and signalling in the ovary 3 DPA. Ethylene is known to induce the senescence of the ovary in pea and *Arabidopsis*. Treatments with the ethylene action inhibitors STS and 2,5-norbornadiene retarded ovary senescence and extended the growth time in which the unpollinated carpel was able to respond to GAs [31]. The loss of ovary response to GAs is also correlated with its senescence and the onset of ovule senescence in *Arabidopsis*[24], suggesting that ovules play a critical role in promoting fruit set in response to GA in *Arabidopsis* unfertilised ovaries. Ethylene produced in the ovules appears to control both the ovule lifespan and the fate of the ovary/fruit [32]. Although we don’t know the ovary tissue where ethylene is produced in zucchini, the growth of unfertilized ovaries decreases at 3 DPA, coinciding with the time at which ovules start to senesce in *Arabidopsis* and pea [24,31], and with the induction of ethylene biosynthesis and signalling genes. Therefore, it is likely that ovule senescence is also the signal that induces the senescence of the whole ovary in zucchini.

**Figure 8:**
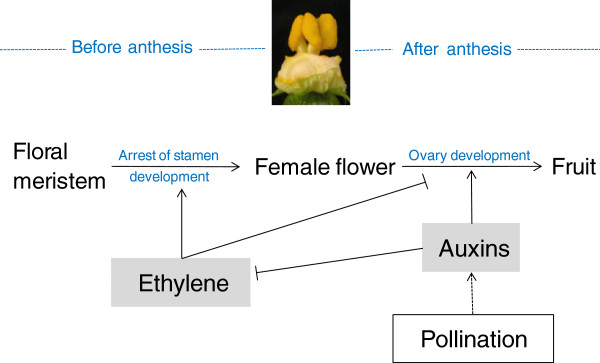
**Ethylene-auxin interactions in the control on sex determination and fruit set in zucchini squash.** During female flower development (before anthesis), ethylene controls the arrest of stamens and the proper maturation of the flower. In the days immediately after anthesis, ethylene regulates negatively fruit set and early fruit development. In fact, a reduction of ethylene in the flower not only induces the conversion of female into bisexual flowers, but also promotes the parthenocarpic development of the fruit. After anthesis, therefore, ethylene production needs to be prevented for a proper development of the ovary and consequently for fruit set and early fruit development. The inhibition of ethylene biosynthesis and signalling in the ovary of zucchini is likely to be caused by auxin, since we have found that auxin regulate negatively ethylene production and signalling during this developmental period. The prevention of ethylene production after pollination is likely to be mediated by auxin, although this has not been proven in this paper (dashed lines).

The low level of ethylene biosynthesis and signalling in pistils at early stages of zucchini fruit development could be coupled with an induction of auxins, one of the main regulators of fruit set in different plant systems [25,33]. We have shown that the application of auxins and TIBA in the flower at anthesis promoted the growth of unfertilized ovaries in the non-parthenocarpic cv. Tosca, concomitantly with a reduction in the production of ethylene and a downregulation of many ethylene biosynthesis and signalling genes. However, the ovaries of Cavili did not respond to auxins and TIBA, suggesting that the parthenocarpy of this cultivar could be caused by an increase in the internal levels of this hormone in the ovary, as has been reported in other species [16,34-36]. Auxins prevented the induction of ethylene at 3 DPA in the two cultivars, which was also correlated with a downregulation of many ethylene biosynthesis and signalling genes in the fruit during the days immediately after anthesis. Taken together these data indicate that auxins in the ovary prevent the biosynthesis and signalling of ethylene during the transition from flower to fruit in zucchini, mimicking pollination and fertilization processes and promoting fruit set and growth (Figure 8). It is known that ethylene and auxins may act synergistically to control certain growth and developmental processes, such as root elongation and root hair formation, but also antagonistically in other processes, such as hypocotyl elongation [37]. As we have shown, in the control of fruit set and early fruit development, ethylene and auxins act antagonistically (Figure 8). The crosstalk of these two hormones in the control of this developmental process in the ovary of *C. pepo* comes into play at the level of ethylene biosynthesis (auxin downregulates *ACS* genes and *CpACO1*) and ethylene response (auxin downregulates ethylene receptor genes, and *CTR*- and EIN3-like genes). These two important plant growth regulators, have wide-ranging and complicated interactions [38]. Auxin has been found to regulate ethylene biosynthesis by controlling the transcription of *ACS* in different plant systems [39,40], but ethylene also regulates auxin biosynthesis in the root meristems of *Arabidopsis*[41,42].

### A reduction in ethylene production and/or response induces parthenocarpy in zucchini squash

The parthenocarpic development of the fruit in the cv. Cavili has been correlated with the development of stamens in female flowers, i.e. a conversion of female into bisexual flowers. Given that ethylene is the main regulator of sex determination in cucurbit species, these data suggest that ethylene is involved in this kind of parthenocarpy. A reduction in ethylene biosynthesis in the pistil, associated with a loss of function mutation in the ethylene biosynthesis genes *CmACS7* and *CsACS2*, is responsible for the conversion of female into bisexual flowers in melon and cucumber, respectively [6,7]. We have shown that the parthenocarpic cv. Cavili produces less ethylene than the non-parthenocarpic cv. Tosca during the days immediately after anthesis, and that parthenocarpy cosegregates with andromonoecy and lower ethylene production in the selfing progeny of Cavili. Moreover, application of ethylene blocking agents at the level of biosynthesis (AVG) or response (STS) induced the conversion of female into bisexual parthenocarpic flowers in *C. pepo*, while ethephon treatments reduced the number of bisexual flowers and therefore the parthenocarpic development of the fruit (Figure 5). Since fruit development in these bisexual flowers occurred when the corolla was still closed, and therefore in absence of pollination/fertilization (Figures 4 and 5), it is likely that a reduction in ethylene production is enough to induce parthenocarpic fruit development in zucchini squash. Therefore it is likely that the parthenocarpy of Cavili is caused by a reduction in ethylene production or response in the post-anthesis female flowers. This reduced ethylene biosynthesis or response could be caused by an increase in auxins, which would also explain the parthenocarpic growth of the fruit. Nevertheless, given that ethylene can also regulate auxin biosynthesis in *Arabidopsis*[41,42], it cannot be ruled out that ethylene regulates auxin biosynthesis negatively during the set and early development of the fruit.

The reduced ethylene production in the ovary of the parthenocarpic fruit of Cavili is not regulated at the level of transcription, since we have shown that the expressions of *ACS* genes do not change between Cavili and Tosca, or were even higher in Cavili for some of the genes analysed, such as *CpACS7*. Therefore, the downregulation of ethylene production in the ovaries of Cavili few days after anthesis appears to be regulated at the post-transcriptional level. Recent findings suggest that post-transcriptional regulation is an important aspect of the control of *ACS* expression, and that the phosphorylation of the conserved C-terminal regions of ACS proteins plays a crucial role in regulating their turnover [43-46].

On the other hand, ethylene is also involved in the maturation and aperture of zucchini female flowers, and the higher ethylene production of these flowers makes the corolla to mature and open faster than in male flowers [4]. Reduced ethylene production and/or response in the female floral buds of Cavili may not only delay the maturation of petals, as we have observed in the bisexual flowers, but also inhibit the induction of ethylene at 3 DPA in absence of pollination. Since the flower is not yet open, the ovary follows normal development reaching the size of a normal fruit but on an immature flower that has not yet reached anthesis. From this point of view, fruit set could not be considered an active transition from the static condition of the ovary in the fully developed flower to the active metabolic condition following pollination and fertilization, but rather the normal programmed pattern of ovary development. Pollination/fertilization prevents ethylene biosynthesis and signalling after anthesis, allowing the fruit to develop normally. In absence of pollination and or fertilization, however, ethylene signal is induced, promoting the abortion of fruit development.

## Conclusions

Figure 8 shows a model for ethylene-auxin interaction on sex determination and fruit set in zucchini squash. Female floral buds of zucchini squash and other cucurbit species produce more ethylene than male flowers [4,8,9]. Before anthesis, ethylene is necessary to arrest the development of stamen and to maintain the sexual identity of female flowers. In this paper we demonstrate that the ethylene produced in the ovary in the days immediately after anthesis plays an important role as a negative regulator of fruit set and early fruit development in zucchini. Pollination/fertilization induces fruit set and development by preventing the production and action of ethylene immediately after anthesis. In absence of pollination/fertilization, ethylene is induced in the ovary, aborting its normal development. Auxins can mimic pollination/fertilization by preventing the induction of ethylene and therefore the abortion of fruit development. Therefore, the effect of pollination/fertilization on ethylene production and signalling in the ovary could be mediated by auxins (Figure 8).

## Methods

### Plants, culture conditions and treatments

Plants of the hybrid cultivars Cavili and Tosca, as well as the F2 segregant generation derived from self-pollination of Cavili, were grown under the same conditions in a greenhouse in Almería (Spain), following standard local commercial practices for both plant nutrition and pest and disease control.

The production of ethylene and the expression of ethylene genes were studied in pollinated and unpollinated ovaries and fruits of the two cultivars at 0, 1, 2, 3, 4 and 5 days post anthesis (DPA). The involvement of auxin in the development of fruits and ethylene production was determined by treating the ovaries of each flower at anthesis with 0.5 ml of 0.8% of the synthetic auxin “fruitone”, consisting of 1-naphthalene acetic acid (NAA, 0.45%) and 1-naphthaleneacetamide (NAA Amide, 1.2%), or with 1 mM triiodobenzoic acid (TIBA), an inhibitor of the polar transport of auxins. The effects of these treatments on early fruit development as well as on the production of ethylene and the expression of ethylene genes were determined in ovaries/fruits over the days immediately after anthesis.

To determine the effect of ethylene in sex determination and parthenocarpy, the apical shoots of the two cultivars were treated every three days for a total of three weeks with 200 ppm ethephon, 1 mM aminoethoxyvinylglycine (AVG), an inhibitor of ethylene biosynthesis, or 0.25 M silver thiosulphate (STS), an inhibitor of ethylene response. Fruit growth rate and sexual expression was determined in control and treated plants of two cultivars: Cavili, which is parthenocarpic and partially andromonoecious, and Tosca, which is non-parthenocarpic and monoecious.

To compare fruit growth and ethylene production between pollinated and non-pollinated fruits, the length and diameter of twelve ovaries/fruits were measured from anthesis to 5 DPA. At the same time ovaries/fruits were harvested at different DPA, and ethylene production was measured on 4 replicates of 3 ovaries/fruits each. For this purpose, three ovaries/fruits at the same developmental stage were enclosed in sealed containers for 6 hours, and the produced ethylene was determined by gas chromatography [4,8]. The production of ethylene of each sample was repeated four times in a VARIAN 3900 gas chromatograph equipped with a flame ionization detector (FID). The same methodology was used to compare fruit growth rate and ethylene production in NAA and TIBA treated ovaries. In the segregant F2 population of Cavili fruit size and ethylene production measurements were only taken at anthesis and at 3 DPA, but using 25 replicates for each flower phenotype, derived from both the 23 monoecious non-parthenocarpic F2 plants and a minimum of 50 partially andromonoecious and parthenocarpic F2 plants.

### Expression analysis by quantitative RT-PCR

Gene expression analysis was performed in three replications per sample. Each replication was the result of an independent extraction of total RNA from 3 different ovaries/fruits. RNA extractions were performed according to the protocol of the *Aurum Total RNA Mini kit* (Biorad). The remains of DNA in RNA samples was eliminated by digestion with *RQ1 RNAse free DNAse* (Promega). Before cDNA synthesis, we verified the absence of DNA in RNA and cDNA samples by PCR amplification with primers for *CpACS27*, a gene which is not expressed in the ovary at anthesis stage or in the fruit of *C. pepo* (unpublished results). cDNA was then synthesized from 600 ng of total RNA using *iScript Reverse Transcription Supermix for RT-qPCR* (Biorad). The expression of genes was evaluated through quantitative RT-PCR by using the *Rotorgene* thermocycler (Qiagen) and *Power SYBR Green PCR Master Mix* (Qiagen). Additional file 1: Table S1 shows the different genes analysed in this paper, including the accession number of their sequences, the corresponding primers used for q-PCR and their putative orthologs in melon and cucumber [47,48]. The q-PCR primers were designed from the 3′ non-coding regions of each gene by using the *Primer Express v 2.0* (Applied Biosystem) software. To avoid possible cross-amplification, and before any q-PCR experiment, the size of the PCR products for each pair of primers was tested in agarose gels, and sequenced. Quantitative RT-PCR reactions consisted of 40 cycles of 20 s at 95°C, 15 s at 59°C and 20 s at 60°C. This was so for all but *CpACS* genes, which were amplified by 40 cycles of 5 s at 95°C and 30 s at 61°C.

Relative expression of each gene was determined by the comparative Ct (*Cycle Threshold*) method using *C. pepo 18S* ribosomal RNA and *ACTIN* genes as internal standards. To use this method, we first demonstrated that the efficiency of amplification for each amplicon was roughly equivalent, regardless of the amount of template cDNA. The absolute value of the slope of ΔCt (Ct of the target gene-Ct of the reference gene) versus serial dilutions of cDNA for a given sample must be less than 0.1. The relative expression of each gene was then calculated relative to a calibrator sample using the formula 2^-ΔΔCt^, where ΔΔCt is the difference between the ΔCt of each sample and the ΔCt of the calibrator sample.

### Statistical analysis

Simple and factorial analyses of variance (ANOVA) at p <0.05 were performed by the Statgraphics Plus v 5.1 software, and each two means were compared with the method of Fisher's least significant difference (LSD). To apply these statistical techniques the variables were required to follow a normal distribution. When the assumption of normality failed, the variables were transformed.

## Abbreviations

ACC: 1-aminocyclopropane-1-carboxylic acid; ACO: Acc oxidase; ACS: Acc synthase; DPA: Days post anthesis; GAs: Gibberellins; 1-MCP: 1- Methylcyclopropene; STS: Silver thiosuphate; AVG: Aminoethoxyvinylglycine; NAA: 1-naphthalene acetic acid; TIBA: Triiodobenzoic acid; CTR: Constitutive triple response; ERS: Ethylene response sensor; ETR: Ethylene receptor; EIN3: Ethylene insensitive3.

## Competing interests

The authors declare that they have no competing interests.

## Authors’ contributions

CM generated the plant material and conducted most of the experiments. SM supervised QPCR experiments and ZM also contributed to them. BP provided *ACS* gene sequences and generated Additional file 1: Table S1. DG and BP revised the document. MJ coordinated the study and drafted the manuscript. All authors read and approved the final manuscript.

## Supplementary Material

Additional file 1: Table S1 Contain a list of genes and q-PCR primers analysed in this paper and their putative orthologs in melon and cucumber [47,48]. Click here for file

Additional file 2: Figure S1 Presents the effect of treatments with ethephon and ethylene inhibitors AVG and STS on sexual expression and parthenocarpy in the cultivars Tosca (A) and Cavili (B). Click here for file
