# Supplementary material for: Involvement of ethylene biosynthesis and signalling in fruit set and early fruit development in zucchini squash (Cucurbita pepo L.)
Source: BMC Plant Biol. 2013 Sep 22;13:139. doi: 10.1186/1471-2229-13-139 (PMC3856489; doi:10.1186/1471-2229-13-139)
Supplement: Additional file 1: Table S1 — Contain a list of genes and q-PCR primers analysed in this paper and their putative orthologs in melon and cucumber [47,48]. [file 1471-2229-13-139-S1.pdf]

Table S1. List of genes and q-PCR primers analysed in this paper. Putative orthologs in melon and cucumber are indicated.

Gene[1]	Reference Accession number	Q-PCR primer sequences	Melon Gene [2]	Cucumber Gene[3]
18S	[9]<br>AF206895	CCGTTGCTCTGATGATTCATGA<br>GTTGATAGGGCAGAAATTTGAATGAT		
ACTINE	[31]<br>HM594170	CCTCTCAATCCCAAAGCTAACAG<br>CGGCCTGGATAGCAACATACA		
CpACS1	[14]<br>AAA33113	TGTCACTGAGCCAGGTTGGTT<br>GCAACGTCAACGGTGTTGTC	MELO3C021182, evalue 0.0<br><br>CM3.5_scaffold00047<br>(319.775-323.449) LGXI	Csa6M006800, evalue 0.0<br><br>Chr6 (576.828-580.112)
CpACS2	[26]<br>CUTC0483191	ACAAGGAGGTAGCCAAGGTTAAG<br>CCCACGACTCAGTTAAACTTTTG	MELO3C016340, evalue 7e-66<br><br>CM3.5_scaffold00027<br>(3.936.912 -3.939.367) LGVII	Csa4M049610, evalue 2e-62<br><br>Chr4 (3.925.814-3.928.351)
CpACS4	[26]<br>CUTC036131	CAGCAGCTTCTCCCATAATTCTA<br>CGTTAATGAATACCTGGAGACGA	MELO3C006840, evalue 0.0<br><br>CM3.5_scaffold00006<br>(6.529.439-6.532.788) LGVI	Csa3M177920, evalue e-176<br><br>Chr3 (12.070.103-2.073.483)
CpACS5	[26]<br>CUTC0383771	TGTGCATCTGAAAGCATAGAAGA<br>TTAGAAGATAGCGACCGAGACAG	MELO3C005597, evalue 0.0<br><br>CM3.5_scaffold00005<br>(6.337.710-6.340.825) LGIX	Csa5M157380, evalue 0.0<br><br>Chr5 (5.575.539-5.578.435)
CpACS6	[26]<br>CUTC0406831	GAAACCAGCTACCAAGAGAAACA<br>TTTTCGTTCGAAGAGTAGATTGC	MELO3C005597, evalue e-122<br><br>CM3.5_scaffold00005<br>(6.337.710-6.340.825) LGIX	Csa5M157380, evalue e-115<br><br>Chr5 (5.575.539-5.578.435)
CpACS7	This paper<br>KC662095	GGTTTAGAGTCGGAGCGATTTAT<br>CTCTCACCAGCATTTTCTGTCTT	MELO3C019008, evalue1e-69<br><br>CM3.5_scaffold00035<br>(3.114.060-3.115.032) LGVII	Csa4M099220, evalue e-122<br><br>Chr4 (6.483.187-6.484.884)
CpACO1	unpublished<br>HQ847860	CATTTGGGACAAAGGTGAGTAACTAC<br>GCTCGGAGACCCTTGATCAA	MELO3C014437, evalue 0.0<br><br>CM3.5_scaffold00022<br>(3.015.704-3.017.224) LGV	Csa6M421630, evalue 0.0<br><br>Chr6 (19.435.577-19.436.822)
CpETR1	unpublished<br>JX014243	AAAGGAGAGCTGCCTGAGAGTC<br>CACGACGCTCTATAAGTTCCGA	MELO3C003906, evalue 0.0<br><br>CM3.5_scaffold00003<br>(56.561-62.893) LGV	Csa2M070880, evalue 8e-65<br><br>Chr2 (5.520.557-5.526.964)
CpERS1	unpublished<br>JX014245	GAGCGTCGGGTTCTATTCGA<br>AACCTGGGATATGCCTTGTATGTTAC	MELO3C003906, evalue 3e-93<br><br>CM3.5_scaffold00003<br>(56.561-62.893) LGV	Csa2M070880, evalue 3e-86<br><br>Chr2 (5.520.557-5.526.964)
CpCTR1	[9]<br>FJ917748	ACTAATGATTAGTTGTGGCTGGATACA<br>AACAGTAATGACATTTCCTAGCAAACA	MELO3C009433, evalue0.0<br><br>CM3.5_scaffold00011<br>(2.117.162-2.125.790) LGIV	Csa3M749850, evalue e-164<br><br>Chr3 (29.127.592-29.136.084)
CpCTR2	[9]<br>FJ917749	ACGTGTTGCTTGGCCTGTTAC<br>TCACTCATGAGTACTGGTTGATTGA	MELO3C009433, evalue 1e-27<br><br>CM3.5_scaffold00011<br>(2.117.162-2.125.790) LGIV	Csa3M749850, evalue e-164<br><br>Chr3 (29.127.592-29.136.084)
CpEIN3.1	This paper<br>KC620381	GCCATCAAATGTTCACAAGACCG<br>AATGAAAAGAGTGCAAGCAGTCTTAC	MELO3C015633, evalue 3e-31<br><br>CM3.5_scaffold00025<br>(3.608.957-3.612.634)	Csa1M601520, evalue 2e-22<br><br>Chr1 (23.382.449-23.384.803)
CpEIN3.2	This paper<br>KC620382	TAGCAGCCAATTCAACCAGTTTAAGCC<br>CGGTAAAGCATCGAATTGAGATCAGG	MELO3C019008, evalue1e-69<br><br>CM3.5_scaffold00035<br>(3.114.060-3.115.032) LGVII	Csa1M601520, evalue 3e-34<br><br>Chr1 (23.382.449-23.384.803)

[1] *Cucurbita pepo* unigenes available at http://www.cucurbigene.net
[2] Best hits of *C. pepo* genes in https://melonomics.net/, database that holds the recently released sequence of the melon genome [47]. Position in the melon genome and in the melon genetic map of each melon unigenes is indicated
[3] Best hits of *C .pepo* genes in http://www.icugi.org/cgi-bin/ICuGI/index.cgi, database that holds the last version of the cucumber genome. Position in the cucumber genome of each gen is indicated [48].
