# Supplementary material for: Involvement of ethylene biosynthesis and signalling in fruit set and early fruit development in zucchini squash (Cucurbita pepo L.)
Source: BMC Plant Biol. 2013 Sep 22;13:139. doi: 10.1186/1471-2229-13-139 (PMC3856489; doi:10.1186/1471-2229-13-139)
Supplement: Additional file 2: Figure S1 — Presents the effect of treatments with ethephon and ethylene inhibitors AVG and STS on sexual expression and parthenocarpy in the cultivars Tosca (A) and Cavili (B). [file 1471-2229-13-139-S2.pdf]

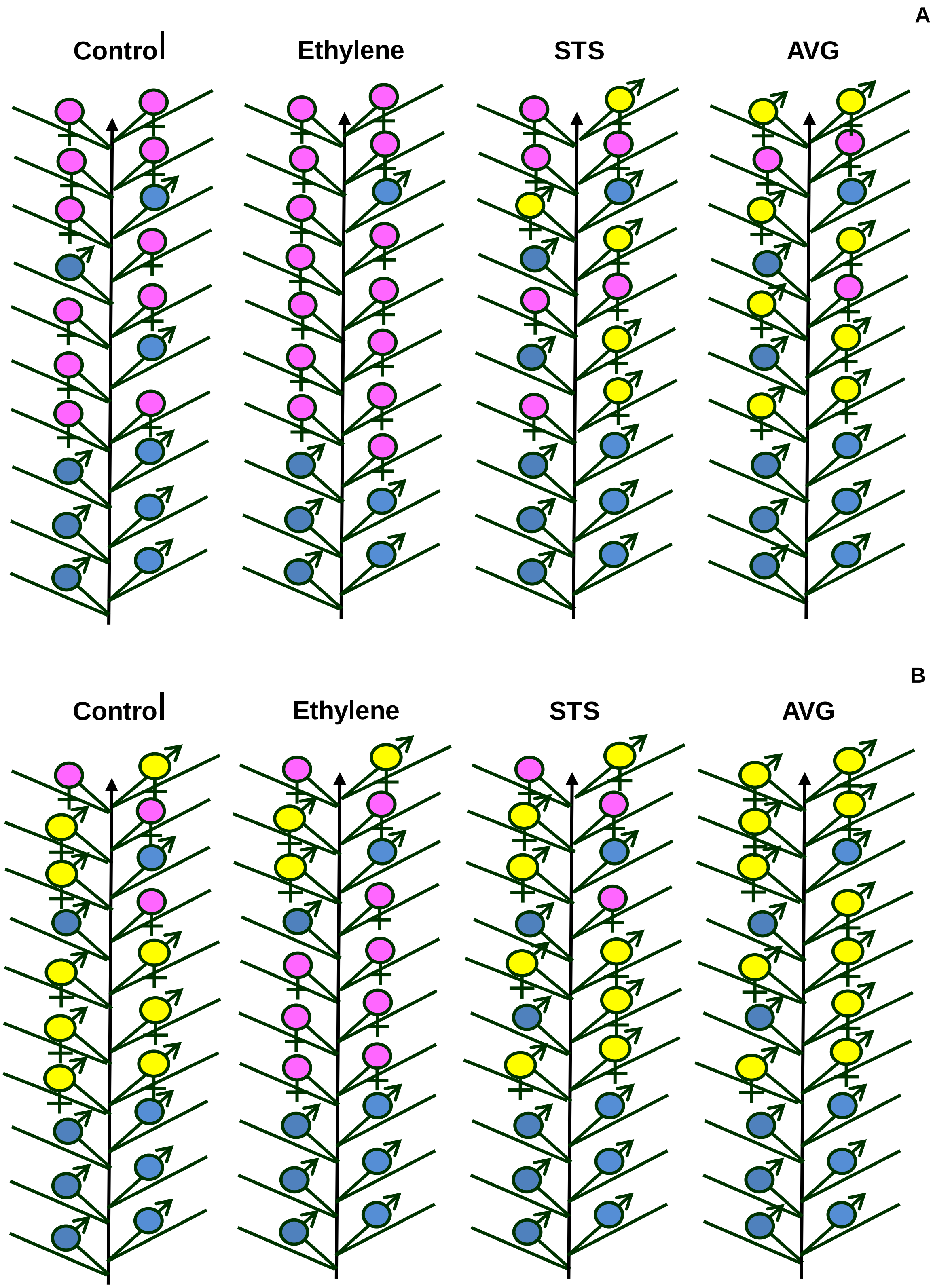

Figure S1: Effect of treatments with ethephon and ethylene inhibitors AVG and STS on sexual expression and parthenocarpy in the cultivars Tosca (A) and Cavili (B). Pink, female flowers; blue, male flowers; yellow, bisexual parthenocarpic flowers.
